# Management and Analysis of Large Qualitative Data Sets: Lessons Learned From Daily Journals for a Behavioral RCT

**DOI:** 10.1093/geroni/igab046.315

**Published:** 2021-12-17

**Authors:** Carol Musil, Alexandra Jeanblanc

**Affiliations:** 1 CWRU School of Nursing, Cleveland, Ohio, United States; 2 Case Western Reserve University, Cleveland, Ohio, United States

## Abstract

As part of a national RCT of a resourcefulness skills training intervention, 342 grandmother caregivers, completed daily online journals for 4 weeks, reporting “challenges or difficulties you faced today with your grandchildren or other family members and how you handled them.” In this paper, we describe the challenges and benefits of using an entirely online design for the distribution and collection of daily journals. We used NVIVO-12 Plus to perform directed content analysis to assess intervention enactment fidelity and compare content between intervention (trained in and prompted to discuss skills) and control groups. Over 92%(n=317) of participants completed daily journals. There was variation in reporting of skill use between groups: 36% of controls spontaneously used the skill “seek professional help” whereas only 4% of the control group reported the skill “change from usual reaction”. With careful management, qualitative data from large samples can be obtained and effectively analyzed for fidelity.

